# Numerical Study on Heat-Transfer Characteristics of Convection Melting in Metal Foam under Sinusoidal Temperature Boundary Conditions

**DOI:** 10.3390/e24121779

**Published:** 2022-12-05

**Authors:** Xiang-Bo Feng, Shi-Fan Huo, Xiao-Tao Xu, Fei Liu, Qing Liu

**Affiliations:** 1 Xi’an Key Laboratory of Advanced Photo-Electronics Materials and Energy Conversion Device, School of Science, Xijing University, Xi’an 710123, China; 2 Shanxi Key Laboratory of Safety and Durability of Concrete Structures, College of Civil Engineering, Xijing University, Xi’an 710123, China; 3 Xi’an Thermal Power Research Institute Co., Ltd., Xi’an 710054, China; 4 Department of Mechanics and Aerospace Engineering, Southern University of Science and Technology, Shenzhen 518055, China; 5 School of Resource Engineering, Xi’an University of Architecture and Technology, Xi’an 710055, China

**Keywords:** convection melting, sinusoidal side wall temperature, lattice Boltzmann method, metal foams, latent heat storage

## Abstract

Convection melting in metal foam under sinusoidal temperature boundary conditions is numerically studied in the present study. A multiple-relaxation-time lattice Boltzmann method, in conjunction with the enthalpy approach, is constructed to model the melting process without iteration steps. The effects of the porosity, phase deviation, and periodicity parameter on the heat-transfer characteristics are investigated. For the cases considered in this work, it is found that the effects of the phase deviation and periodicity parameter on the melting rate are weak, but the melting front can be significantly affected by the sinusoidal temperature boundary conditions.

## 1. Introduction

Latent heat storage (LHS), which uses solid–liquid phase-change materials (PCMs) as thermal-energy storage media, has been widely employed in industrial waste heat utilization to build energy saving systems, solar thermal utilization systems, etc. LHS with solid–liquid PCMs has become an important research topic during the past 30 years, and numerous reviews about this topic have been published. Zalba et al. [1] carried out a comprehensive review of the materials, the heat transfer process, and applications of LHS using solid–liquid PCMs. Farid et al. [2] reviewed the efforts in developing new PCMs for LHS applications. In a recent review by Nazir et al. [3], the applications of various PCMs, based on their thermophysical properties, were summarized, and the strategies for improving the characteristics of thermal-energy storage through nanomaterial additives, as well as encapsulation, were discussed in detail.

LHS with the use of solid–liquid PCMs has gradually become the preferred thermal-energy storage pattern, as solid–liquid PCMs have some outstanding features, such as the energy storage density being very high and the temperature fluctuation being small. However, the thermal conductivities for most of the solid–liquid PCMs are low (0.1~0.6 W/(m·K) [4]). This serious shortcoming strongly slows down the charging and discharging rates of thermal energy. To improve the LHS system’s thermal performance, three main kinds of enhancement approaches have been employed: improving the uniformity of heat-transfer process, enhancing the thermal conductivity of PCMs, and extending the heat-transfer surface [5]. Among these enhancement approaches, enhancing the thermal conductivity performance of PCMs is an efficient way to improve the LHS system’s thermal performance. High-porosity metal foams attract great attention for LHS applications because of their attractive advantages, such as high thermal conductivity and large specific surface areas. 

In recent decades, numerous numerical studies on the characteristics of solid–liquid phase change in metal foams (porous media) have been performed. Weaver and Viskanta [6] numerically and experimentally investigated the melting process of ice in a cylindrical capsule filled with glass or aluminum beads. Beckermann and Viskanta [7] studied the melting and solidification processes of gallium in a square cavity filled with glass beads. They found that the shape of the interface can be considerably influenced by the convection effect in the liquid region. Tong et al. [8] performed a numerical study on the melting and freezing of a water–aluminum matrix system in a cylindrical annulus. They found that the heat-transfer rates of enhanced cases were increased by one order of magnitude, compared with that of the base case without an aluminum matrix.

In the numerical studies [6,7,8], the local thermal equilibrium (LTE) assumption is adopted, as the thermal conductivity of the solid matrix is low. However, for high-thermal-conductivity metal foams, such as copper or aluminum foam, the local thermal non-equilibrium (LTNE) effect (temperature difference) between a PCM and a metal matrix during the melting process should be considered. Harris et al. [9] developed an approximate theoretical enthalpy model (LTNE model) in which a temperature difference between the PCM and the walls of the pores was maintained. Based on the approximate model, the conditions for the occurrence of LTE were analyzed. Mesalhy et al. [10] developed a two-temperature model to analyze the LTNE effect between the PCM and the metal matrix, and a parametric study was performed to investigate the effects of thermal conductivity and porosity. Krishnan et al. [11] also proposed an LTNE model for simulating convection melting in metal foams, and the merits of using metal foam for enhancing thermal storage systems’ effective thermal conductivity were discussed. An LTNE model regarding the volume change of the PCM was proposed by Yang and Garimella [12], and the effects of volume expansion/shrinkage were analyzed. Li et al. [13] investigated the melting of paraffin embedded in open-cell copper foam, and the effects of the morphology parameters of the metal foam on the temperature distributions were investigated. Zhao et al. [14] performed a numerical investigation on melting and solidification in copper foam, and the kinetic undercooling of solidification was analyzed. Wang et al. [15] studied the pore-scale melting in metal foams; different metal foams combined with paraffin and other PCMs were investigated to obtain the composite materials’ effective thermal conductivity.

The above literature review indicates that many numerical studies have been carried out on the heat-transfer performance of PCMs in metal foams based on an LTNE model. Moreover, our literature survey with respect to improving an LHS system’s thermal performance using metal foams found that nearly all of the numerical studies were conducted under constant wall heat flux or constant wall temperatures (uniform thermal boundary conditions). A fundamental understanding of the heat-transfer characteristics of melting in metal foams under non-uniform thermal boundary conditions is still lacking, and more studies are required. For natural convection in enclosures, previous studies indicated that non-uniform thermal boundary conditions (e.g., sinusoidal temperature boundary conditions) can significantly affect the flow structures and heat-transfer characteristics [16,17,18]. As expected, new heat-transfer characteristics can be created in the solid–liquid phase change of PCMs under sinusoidal temperature boundary conditions. Hence, this work aimed to study the heat-transfer characteristics of convection melting in metal foam under sinusoidal temperature boundary conditions. A multiple-relaxation-time (MRT) lattice Boltzmann (LB) method, in conjunction with the enthalpy approach, was constructed to model the melting process without iteration steps. This work will help in providing a valuable reference for improving the thermal performance of LHS systems.

## 2. Model Description

### 2.1. Physical Model

**Figure 1:**
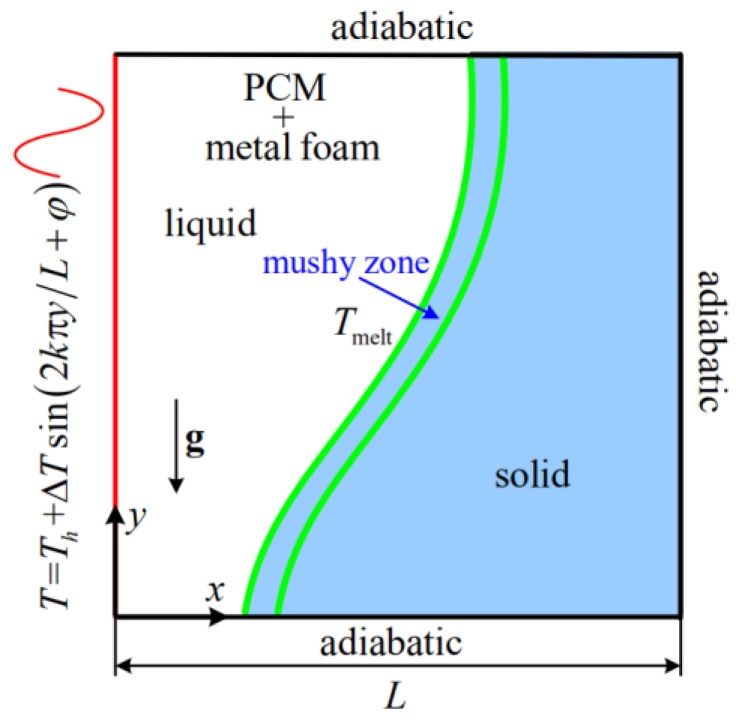
Physical model.

The problem considered in this work is shown in Figure 1. Initially, the temperatures of the PCM and metal foam are equal to $T_{i}$ ($T_{i}<T_{\mathrm{melt}}$). At t=0, a sinusoidally varying temperature $T=T_{h}+\Delta T\sin\left(2k\pi y/L+\varphi \right)$ ($T_{h}>T_{\mathrm{melt}}$) is imposed on the left wall, and then the PCM begins to melt. Note that the average temperature of the left wall is $T_{h}$, $\Delta T=T_{h}-T_{\mathrm{melt}}$ is the characteristics temperature, k is the periodicity parameter, and φ is the phase deviation (phase of the sinusoidal profile).

### 2.2. Governing Equations

For convection melting of solid–liquid PCMs embedded in metal foams, the following assumptions are made: (1) metal foam (m) is homogeneous and the pore diameter is uniform; (2) the flow (liquid region) is incompressible and laminar; (3) the volume change is neglected, i.e., $\rho_{f}\;=\;\rho_{l}=\;\rho_{s}$ (the subscript f denotes PCM, l denotes liquid PCM and s denotes solid PCM). Based on the LTNE model, the governing equations are provided by [11,19,20,21]

(1) ∇⋅u=0

(2) $$\frac{\partial \;u}{\partial \;t}+\left(u\cdot \nabla \right)\left(\frac{u}{\phi }\right)=-\frac{1}{\rho_{f}}\nabla \left(\phi p\right)+v_{e}\nabla^{2}u+F$$

(3) $$\frac{\partial }{\partial \;t}\left(\phi \rho_{f}c_{pf}T_{f}\right)+\nabla \cdot \left(\rho_{l}c_{pl\;}T_{f}u\right)=\nabla \cdot \left(k_{ef}\nabla T_{f}\right)+h_{mf}a_{mf}\left(T_{m}-T_{f}\right)-\frac{\partial }{\partial t}\left(\phi \rho_{l}L_{a}f_{l}\right)$$

(4) $$\frac{\partial }{\partial \;t}\left[\left(1-\phi \right)\rho_{m}c_{pm\;}T_{m}\right]=\nabla \cdot \left(k_{em}\nabla T_{m}\right)+h_{mf}a_{mf}\left(T_{f}-T_{m}\right)$$

where **u** and p are the velocity and pressure in the liquid region, respectively; $\rho_{f}$ is the density; T is the temperature; ϕ is the metal foam’s porosity; $c_{p}$ is the specific heat; $v_{e}$ is the effective kinematic viscosity; $k_{e}$ is the effective thermal conductivity; $h_{mf}$ is the interfacial heat-transfer coefficient; $a_{mf}$ is the specific surface area of metal matrix; $f_{l}$ is the liquid fraction; and $L_{a}$ is the PCM’s latent heat.

The total body force F is determined by [22,23]

(5) $$F=-\frac{\phi v}{K}u-\frac{\phi F_{\phi }}{\sqrt{K}}\left|u\right|u+\phi \;G$$

where v is the liquid PCM’s kinematic viscosity and K and $F_{\phi }$ are the metal foam’s permeability and inertia coefficient, respectively. G is the buoyancy force approximated by

(6) $$G=-g\beta \left(T_{f}-T_{0}\right)f_{l}$$

where g is the gravitational acceleration, $T_{0}$ is the reference temperature, and β is the thermal-expansion coefficient.

For metal foams (e.g., aluminum or copper foam), the correlations of $F_{\phi }$ and K can be found in [24,25]. The effective thermal conductivities $k_{ef}$ and $k_{em}$ can be determined by analytical models [25,26]. In previous studies, the correlation for convection heat transfer through a bank of staggered cylinders proposed by Churchill and Chu [27] was widely employed to determine $h_{mf}$. The empirical formula for $a_{mf}$ can be found in [25]. To determine the temperature-dependent (thermodynamic or dynamic mechanical) properties of complex materials, such as the cross-linking of polymers, the methods proposed by Likozar and Krajnc [28,29,30] can be employed.

## 3. Numerical Method

As a mesoscopic approach evolved from the lattice-gas automata [31], the LB method [32,33,34] has become an efficient numerical methodology for modeling solid–liquid phase-change problems [35,36]. In this section, the MRT-LB method, in conjunction with the enthalpy approach, is introduced to model the melting process without iteration steps.

### 3.1. MRT-LB Equation for Flow Field

For the 2D problem considered in this work, the D2Q9 lattice is employed [34]

(7) $$e_{i}=\{\begin{array}{ll} \left(0,0\right), & i=0\\ \left(\cos\left[\left(i-1\right)\pi /2\right],\sin\left[\left(i-1\right)\pi /2\right]\right)c, & i=1\sim 4\\ \left(\cos\left[\left(2i-9\right)\pi /4\right],\sin\left[\left(2i-9\right)\pi /4\right]\right)\sqrt{2}c, & i=5\sim 8 \end{array}$$

where $c=\delta_{x}/\delta_{t}$ is the lattice speed ($\delta_{x}$ is the lattice step and $\delta_{t}$ is the time step). In this work, c is set to 1 ($\delta_{x}=\delta_{t}$).

The MRT-LB equation for the flow field can be written as [37,38,39]

(8) $$f_{i}\left(x+e_{i}\delta_{t},\;t+\delta_{t}\right)=f_{i}\left(x,\;t\right)-{\bar{\Lambda }}_{ij}\left(f_{j}-f_{j}^{eq}\right)|{}_{\left(x,\;t\right)}+\delta_{t}\left({\tilde{S}}_{i}-0.5{\bar{\Lambda }}_{ij}{\tilde{S}}_{j}\right)$$

where $f_{i}\left(x,\;t\right)$ is the density distribution function, $f_{i}^{eq}\left(x,\;t\right)$ is the equilibrium value of $f_{i}\left(x,\;t\right)$, $\bar{\Lambda }=[{\bar{\Lambda }}_{ij}]$ is the collision matrix, and ${\tilde{S}}_{i}$ is the forcing term.

The MRT-LB Equation (8) can be divided into two parts: a collision part and a streaming part. By multiplying a transformation matrix M, the collision part can be carried out in moment space as

(9) $$m^{*}\left(x,\;t\right)=m\left(x,\;t\right)-\Lambda \left(m-m^{eq}\right)|{}_{\left(x,\;t\right)}+\delta_{t}\left(I-\frac{\Lambda }{2}\right)S$$

The streaming part is performed in velocity space as

(10) $$f_{i}\left(x+e_{i}\delta_{t},\;t+\delta_{t}\right)=f_{i}^{*}\left(x,\;t\right)$$

where Λ is the relaxation matrix ($\Lambda =M\bar{\Lambda }M^{-1}=\mathrm{diag}\left(1,\;1,\;1,s_{e},\;s_{v},\;s_{v},\;s_{q},\;s_{q},s_{\varepsilon }\right)$), m=|m〉=Mf, $m^{eq}=|m_{}^{eq}\rangle =Mf^{eq}$, $S=|S\rangle =M\tilde{S}$, in which f=|f〉, $f^{eq}=|f_{}^{eq}\rangle$, and $\tilde{S}=|\tilde{S}\rangle$. Here, Dirac notation |⋅〉 denotes a nine-dimensional column vector, e.g., $|m\rangle ={\left(m_{0},m_{1},\ldots \;,\;m_{8}\right)}^{T}$. $f_{i}^{*}$ is determined by $f^{*}=|f_{}^{*}\rangle =M^{-1}m^{*}$.

M is a non-orthogonal transformation matrix [39]

(11) $$M=\left[\begin{array}{rrrrrrrrr} 1 & 1 & 1 & 1 & 1 & 1 & 1 & 1 & 1\\ 0 & 1 & 0 & -1 & 0 & 1 & -1 & -1 & 1\\ 0 & 0 & 1 & 0 & -1 & 1 & 1 & -1 & -1\\ 0 & 1 & 1 & 1 & 1 & 2 & 2 & 2 & 2\\ 0 & 1 & -1 & 1 & -1 & 0 & 0 & 0 & 0\\ 0 & 0 & 0 & 0 & 0 & 1 & -1 & 1 & -1\\ 0 & 0 & 0 & 0 & 0 & 1 & 1 & -1 & -1\\ 0 & 0 & 0 & 0 & 0 & 1 & -1 & -1 & 1\\ 0 & 0 & 0 & 0 & 0 & 1 & 1 & 1 & 1 \end{array}\right]$$

The equilibrium moments $\left\{m_{i}^{eq}\right\}$ are determined by

(12) $$m_{0}^{eq}=\rho_{f},\;m_{1}^{eq}=\rho_{f}u_{x},\;m_{2}^{eq}=\rho_{f}u_{y},\;m_{3}^{eq}=\frac{2}{3}\rho_{f}+\frac{\rho_{f}\left(u_{x}^{2}+u_{y}^{2}\right)}{\phi },\;m_{4}^{eq}=\frac{\rho_{f}\left(u_{x}^{2}-u_{y}^{2}\right)}{\phi }\;m_{5}^{eq}=\rho_{f}u_{x}^{}u_{y},\;m_{6}^{eq}=\frac{1}{3}\rho_{f}u_{y},\;m_{7}^{eq}=\frac{1}{3}\rho_{f}u_{x},\;m_{8}^{eq}=\frac{1}{9}\rho_{f}+\frac{1}{3}\frac{\rho_{f}\left(u_{x}^{2}+u_{y}^{2}\right)}{\phi }$$

The source terms $\left\{S_{i}^{}\right\}$ are determined by

(13) $$S_{0}=0,\;S_{1}=\rho_{f}F_{x},\;S_{2}=\rho_{f}F_{y},\;S_{3}=\frac{2\rho_{f}\left(u_{x}F_{x}+u_{y}F_{y}\right)}{\phi },\;S_{4}=\frac{2\rho_{f}\left(u_{x}F_{x}-u_{y}F_{y}\right)}{\phi }\;S_{5}=\frac{\rho_{f}\left(u_{x}F_{y}+u_{y}F_{x}\right)}{\phi },\;S_{6}=\frac{1}{3}\rho_{f}F_{y},\;S_{7}=\frac{1}{3}\rho_{f}F_{x},\;S_{8}=\frac{2}{3}\frac{\rho_{f}\left(u_{x}F_{x}+u_{y}F_{y}\right)}{\phi }$$

To implement the non-slip velocity boundary condition on the phase interface accurately, the volumetric LB scheme [40] is employed; then, a new density distribution function is defined:

(14) $$f_{i}^{+}=f_{l}f_{i}+\left(1-f_{l}\right)f_{i}^{eq}\left(\rho_{f},u_{s}\right)$$

In Equation (14), the superscript “+” denotes that the solid-phase effect has been considered, and $u_{s}\;=0$ (the solid phase is static). Accordingly, $\rho_{f}$ and u are defined as

(15) $$\rho_{f}=\sum_{i=0}^{8}f_{i}^{}$$

(16) $$\rho_{f}u=\sum_{i=0}^{8}e_{i}f_{i}^{+}+\frac{\delta_{t}}{2}\rho_{f}F$$

p is defined as $p=\rho c_{s}^{2}/\phi$ ($c_{s}=1/\sqrt{3}$ is the sound speed). Explicitly, u can be calculated via [41]

(17) $$u=\frac{v}{l_{0}+\sqrt{l_{0}^{2}+l_{1}\left|v\right|}}$$

where

(18) $$\rho_{f}v=\sum_{i=0}^{8}e_{i}f_{i}^{+}+\frac{\delta_{t}}{2}\rho_{f}\phi G$$

(19) $$l_{0}=\frac{1}{2}\left(1+\phi \frac{\delta_{t}}{2}\frac{v}{K}\right),\;l_{1}=\phi \frac{\delta_{t}}{2}\frac{F_{\phi }}{\sqrt{K}}$$

The kinetic viscosity $v=c_{s}^{2}\left(s_{v}^{-1}-0.5\right)\delta_{t}$ and the bulk viscosity $\xi =c_{s}^{2}\left(s_{e}^{-1}-0.5\right)\delta_{t}$.

### 3.2. MRT-LB Equation for the Temperature Field of the PCM

Equation (3) can be rewritten as

(20) $$\frac{\partial H_{f}}{\partial \;t}+\nabla \cdot \left(\frac{c_{pl\;}T_{f}u}{\phi }\right)=\nabla \cdot \left(\frac{k_{ef}}{\phi \rho_{l}}\nabla T_{f}\right)+\frac{h_{mf}a_{mf}\left(T_{m}-T_{f}\right)}{\phi \rho_{l}}$$

where $H_{f}=\sigma c_{pl\;}T_{f}+L_{a}f_{l}$ is the effective enthalpy and $\sigma =\frac{\rho_{f}c_{pf}}{\rho_{l}c_{pl\;}}=\frac{f_{l}\rho_{l}c_{pl}+\left(1-f_{l}\right)\rho_{s}c_{ps}}{\rho_{l}c_{pl\;}}$ is the heat-capacity ratio. When $f_{l}=1$ (liquid region), $H_{f}=c_{pl\;}T_{f}+L_{a}$ and $\sigma_{l}=1$; when $f_{l}=0$ (solid region), $H_{f}=\sigma_{s}c_{pl\;}T_{f}$ and $\sigma_{s}=\frac{\rho_{s}c_{ps}}{\rho_{l}c_{pl\;}}$.

For the temperature field of the PCM, governed by Equation (20), the D2Q5 lattice is adopted and $\left\{e_{i}|i=0,\;\ldots ,\;4\right\}$ are provided in Equation (7). The enthalpy-based MRT-LB equation is determined by

(21) $$g\left(x+e\delta_{t},\;t+\delta_{t}\right)=g\left(x,\;t\right)-N^{-1}\Theta \left(n_{g}-n_{g}^{eq}\right)|{}_{\left(x,\;t\right)}+\delta_{t}N^{-1}S_{\mathrm{PCM}}$$

where $g_{i}$ is the enthalpy distribution function and $\Theta =\mathrm{diag}\left(1,\;\zeta_{\alpha },\zeta_{\alpha },\zeta_{e},\zeta_{e}\right)$ is the relaxation matrix.

Through the transformation matrix N, the collision part of the MRT-LB Equation (21) is carried out in moment space as

(22) $$n_{g}^{*}\left(x,\;t\right)=n_{g}\left(x,\;t\right)-\Theta \left(n_{g}-n_{g}^{eq}\right)|{}_{\left(x,\;t\right)}+\delta_{t}S_{\mathrm{PCM}}$$

The streaming part is performed in velocity space as

(23) $$g_{i}\left(x+e_{i}\delta_{t},\;t+\delta_{t}\right)=g_{i}^{*}\left(x,\;t\right)$$

where $n_{g}\;=Ng$ is the moment, and $n_{g}^{eq}\;=Ng^{eq}$ is the corresponding equilibrium moment. Here, $g_{i}^{eq}$ is the equilibrium value of $g_{i}$, and $g^{*}\;=N^{-1}n_{g}^{*}$.

N is a non-orthogonal transformation matrix [39]

(24) $$N=\left[\begin{array}{rrrrr} 1 & 1 & 1 & 1 & 1\\ 0 & 1 & 0 & -1 & 0\\ 0 & 0 & 1 & 0 & -1\\ 0 & 1 & 1 & 1 & 1\\ 0 & 1 & -1 & 1 & -1 \end{array}\right]$$

The equilibrium moment $n_{g}^{eq}$ is

(25) $$n_{g}^{eq}={\left(H_{f},\frac{c_{pl\;}T_{f}u_{x}}{\phi },\frac{c_{pl\;}T_{f}u_{y}}{\phi },\varpi_{1}c_{pl\;}T_{f}\;,0\right)}^{T}$$

where $\varpi_{1}\in \left(0,\;1\right)$. Correspondingly, $g_{i}^{eq}$ is given by

(26) $$g_{i}^{eq}=\{\begin{array}{ll} H_{f}-\varpi_{1}c_{pl\;}T_{f}, & i=0\\ \frac{1}{4}\varpi_{1}c_{pl\;}T_{f}\left(1+\frac{e_{i}\cdot u}{c_{sf}^{2}\phi }\right), & i=1\sim 4 \end{array}$$

where $c_{sf}^{}=\sqrt{\varpi_{1}/2}$ is the sound speed.

The source term $S_{\mathrm{PCM}}$ is chosen as

(27) $$S_{\mathrm{PCM}}=S_{\mathrm{PCM}}{\left(1,0,\;0,0,0\right)}^{T}$$

where $S_{\mathrm{PCM}}=Sr_{f}\;+\frac{1}{2}\delta_{t}\partial_{t}Sr_{f}$ and $Sr_{f}=h_{mf}a_{mf}\left(T_{m}-T_{f}\right)/\left(\phi \rho_{l}\right)$.

$H_{f}$ is defined as

(28) $$H_{f}=\sum_{i=0}^{4}g_{i}$$

$T_{f}$ can be determined via the following equation:

(29) $$T_{f}=\{\begin{array}{ll} H_{f}/\left(\sigma_{s}c_{pl}\right), & H_{f}\leq H_{fs}\\ T_{fs}+\frac{H_{f}-H_{fs}}{H_{fl}-H_{fs}}\left(T_{fl}-T_{fs}\right), & H_{fs}<H_{f}<H_{fl}\\ T_{fl}+\left(H_{f}-H_{fl}\right)/\left(\sigma_{l}c_{pl}\right), & H_{f}\geq H_{fl} \end{array}$$

where $T_{fs}$ is solidus temperature and $T_{fl}$ is liquidus temperature ($T_{fs}\leq T_{fl}$); $H_{fs}$ ($H_{fl}$) is the effective enthalpy corresponding to $T_{fs}$ ($T_{fl}$).

$f_{l}$ is determined by

(30) $$f_{l}=\{\begin{array}{ll} 0, & H_{f}\leq H_{fs}\\ \frac{H_{f}-H_{fs}}{H_{fl}-H_{fs}}, & H_{fs}<H_{f}<H_{fl}\\ 1, & H_{f}\geq H_{fl} \end{array}$$

$\alpha_{ef}$ is defined as

(31) $$\alpha_{ef}=\frac{k_{ef}}{\phi \rho_{l}c_{pl}}=c_{sf}^{2}\left(\zeta_{\alpha }^{-1}-\frac{1}{2}\right)\delta_{t}$$

### 3.3. MRT-LB Equation for the Temperature Field of Metal Foam

Equation (4) can be rewritten as

(32) $$\frac{\partial \left(c_{pm\;}T_{m}\right)}{\partial \;t}=\nabla \cdot \left(\frac{k_{em}}{\left(1-\phi \right)\rho_{m}}\nabla T_{m}\right)+\frac{h_{mf}a_{mf}\left(T_{f}-T_{m}\right)}{\left(1-\phi \right)\rho_{m}}$$

For the temperature field of metal foam, governed by Equation (32), the MRT-LB equation based on D2Q5 lattice is as follows:

(33) $$h\left(x+e\delta_{t},\;t+\delta_{t}\right)=h\left(x,\;t\right)-N^{-1}Q\left(n_{h}-n_{h}^{eq}\right)|{}_{\left(x,\;t\right)}+\delta_{t}N^{-1}S_{\mathrm{metal}}$$

where $h_{i}\left(x,\;t\right)$ is the temperature distribution function, $Q=\mathrm{diag}\left(1,\eta_{\alpha },\;\eta_{\alpha },\;\eta_{e},\;\eta_{e}\right)$ is the relaxation matrix, and N is given by Equation (24). 

The collision part of the MRT-LB Equation (33) is performed in moment space as

(34) $$n_{h}^{*}\left(x,\;t\right)=n_{h}\left(x,\;t\right)-Q\left(n_{h}-n_{h}^{eq}\right)|{}_{\left(x,\;t\right)}+\delta_{t}S_{\mathrm{metal}}$$

where $n_{h}\;=Nh$ is the moment, and $n_{h}^{eq}\;=Nh^{eq}$ is the corresponding equilibrium moment. Here, $h_{i}^{eq}$ is the equilibrium value of $h_{i}$. The streaming step is carried out in the velocity space as follows:

(35) $$h_{i}\left(x+e_{i}\delta_{t},\;t+\delta_{t}\right)=h_{i}^{*}\left(x,\;t\right)$$

where $h^{*}\;=N^{-1}n_{h}^{*}$. The equilibrium moment $n_{h}^{eq}$ is defined as

(36) $$n_{h}^{eq}={\left(c_{pm}T_{m},0,0,\varpi_{2}c_{pm}T_{m},0\right)}^{T}$$

where $\varpi_{2}\in (0,1)$. $h_{i}^{eq}$ is determined by

(37) $$h_{i}^{eq}=\{\begin{array}{ll} (1-\varpi_{2})c_{pm}T_{m}, & \;i=0\\ \frac{1}{4}\varpi_{2}c_{pm}T_{m}, & \;i=1\sim 4 \end{array}$$

The source term $S_{\mathrm{metal}}$ is chosen as

(38) $$S_{\mathrm{metal}}=S_{\mathrm{metal}}{\left(1,0,\;0,0,0\right)}^{T}$$

where $S_{\mathrm{metal}}=Sr_{m}\;+\frac{1}{2}\delta_{t}\partial_{t}Sr_{m}$ and $Sr_{m}=h_{mf}a_{mf}\left(T_{f}-T_{m}\right)/\left[\left(1-\phi \right)\rho_{m}\right]$.

$T_{m}$ is defined by

(39) $$T_{m}=\frac{1}{c_{pm}}\sum_{i=0}^{4}h_{i}$$

$\alpha_{em}$ is given by

(40) $$\alpha_{em}=\frac{k_{em}}{\left(1-\phi \right)\rho_{m}c_{pm}}=c_{sm}^{2}\left(\eta_{\alpha }^{-1}-\frac{1}{2}\right)\delta_{t}$$

where $c_{sm}=\sqrt{\varpi_{2}/2}$ is the sound speed.

## 4. Numerical Results

In this section, numerical simulations are performed to investigate the effects of the porosity, the phase deviation, and the periodicity parameter on the heat-transfer performance of the convection melting of solid–liquid PCM embedded in metal foam under sinusoidal temperature boundary conditions. The characteristic parameters include $Pr=v_{fl}/\alpha_{fl}$ (Prandtl number), $Ra=g\beta \Delta TL^{3}/\left(v_{fl}\alpha_{fl}\right)$ (Rayleigh number), $Da=K/L^{2}$ (Darcy number), $J=v_{e}/v_{fl}$ (viscosity ratio), $\lambda =k_{m}/k_{fl}$ (thermal conductivity ratio), $\Gamma =\alpha_{m}/\alpha_{fl}$ (thermal diffusivity ratio), $\hat{\sigma }=\rho_{m}c_{pm}/\left(\rho_{l}c_{pl}\right)$ (metal foam to liquid PCM heat capacity ratio), $H_{v}=h_{mf}a_{mf}d_{p}^{2}/k_{f}$ (volumetric heat transfer coefficient), $Fo=t\alpha_{fl}/L^{2}$ (Fourier number), and $St=c_{pl}\Delta T/L_{a}$ (Stefan number), where $\alpha_{f}=k_{f}/{\left(\rho c_{p\;}\right)}_{f}$ is thermal diffusivity of PCM, $\alpha_{m}=k_{m}/{\left(\rho c_{p\;}\right)}_{m}$ is thermal diffusivity of metal foam, and $d_{p}^{}$ is mean pore diameter.

**Figure 2:**
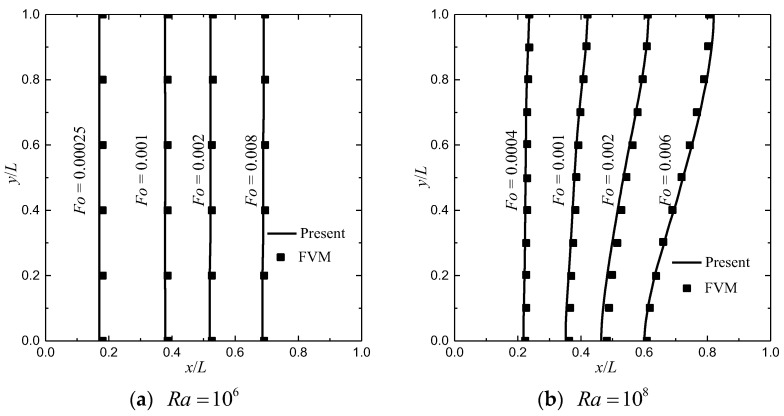
The melting front ($f_{l}=0.5$) at different *Fo*.

**Figure 3:**
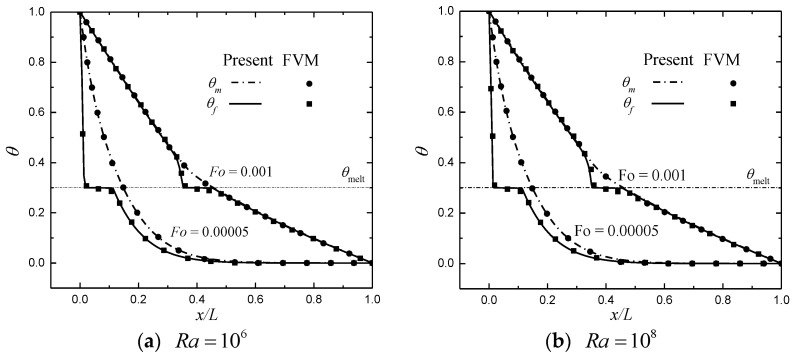
Temperature profiles (y/L=0.5) at different *Fo*.

In simulations, the required parameters are chosen as follows: Pr=50, $F_{\phi }=0.068$, $Da=10^{-4}$, St=1, $\delta_{x}=\delta_{y}=\delta_{t}=1$ (c=1), $c_{pl}=c_{ps}\;=1$, $J=\hat{\sigma }=1$, $H_{v}=5.9$, $\lambda =\Gamma =10^{3}$, $d_{p}/L=0.0135$, $k_{f}=0.0005$ and $\varpi_{1}=\varpi_{2}=1/2$. The relaxation rate $\zeta_{e}$ is determined by $\zeta_{e}\;=2-\zeta_{\alpha }$ to reduce the unphysical numerical diffusion [21,42]. The non-equilibrium extrapolation scheme [43] is adopted to realize the velocity and thermal boundary conditions. Numerical simulations are performed based on a grid size of $N_{x}\times N_{y}=150\times 150$. First, comparisons between the results predicted by the finite-volume method (FVM) [11] and the present method are made to validate the reliability of the present method. The predicted results are shown in Figure 2 and Figure 3, where the melting front (solid–liquid interface) and temperature profiles ($\theta =\left(T-T_{melt}\right)/\Delta T$) at different times for $Ra=10^{6}$ and $10^{8}$ with ϕ=0.8 are presented. In the figures, it can be seen that the present results match well with the results in [11]. In what follows, the effects of the porosity, the phase deviation, and the periodicity parameter on the heat-transfer performance are investigated.

### 4.1. Effects of the Porosity and Phase Deviation

**Figure 4:**
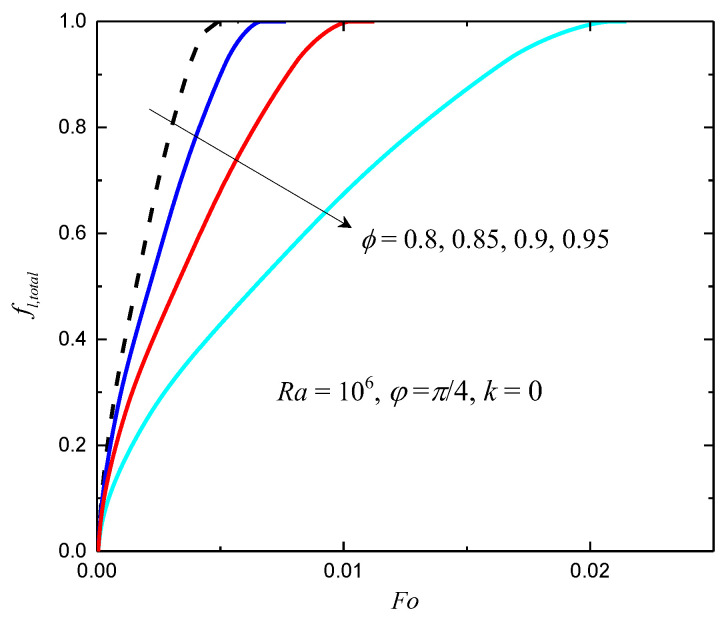
The total liquid fractions for different ϕ with *Ra* = 10^6^, *φ* = π/4 and *k* = 0.

In this subsection, the effects of the porosity and the phase deviation are investigated. In Figure 4, the total liquid fractions for different ϕ with $Ra=10^{6}$, φ=π/4 and k=0 are shown. As can be seen in Figure 4, the melting rate decreases as ϕ increases. When ϕ=0.8, the completely melting time Fo=0.00485. As ϕ increases to 0.9 and 0.95, the completely melting time Fo augments to 0.0103 and 0.0209, respectively. When ϕ increases from 0.8 to 0.9, the completely melting time increases by 112.37%; when ϕ increases from 0.9 to 0.95, the completely melting time increases by 102.91%. The influence of the porosity on the melting rate is induced by two factors: one is that the mass of the metal foam decreases as the porosity increases, which reduces the effective thermal conductivity, and consequently, the performance of heat transfer is deteriorated; the other is that the mass of the PCM increases as the porosity increases, which results in melting-time augmentation.

**Figure 5:**
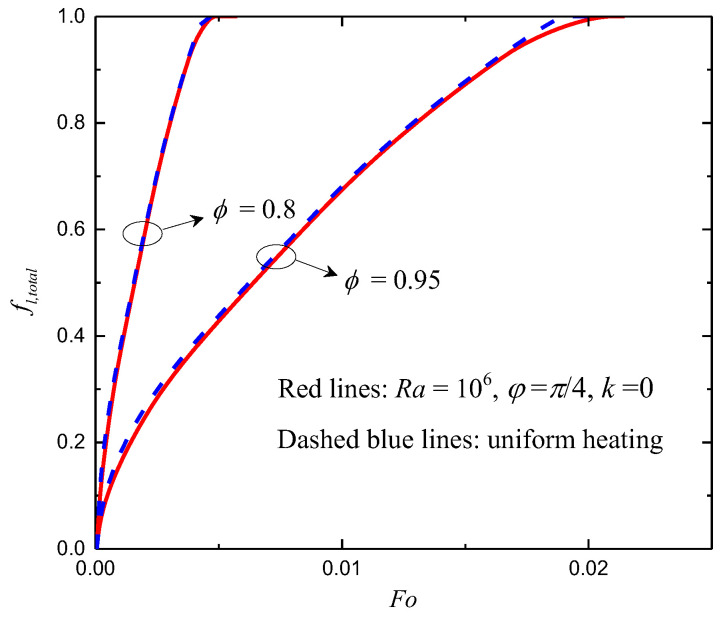
The total liquid fractions for ϕ=0.8 and 0.95 under non-uniform and uniform thermal boundary conditions.

**Figure 6:**
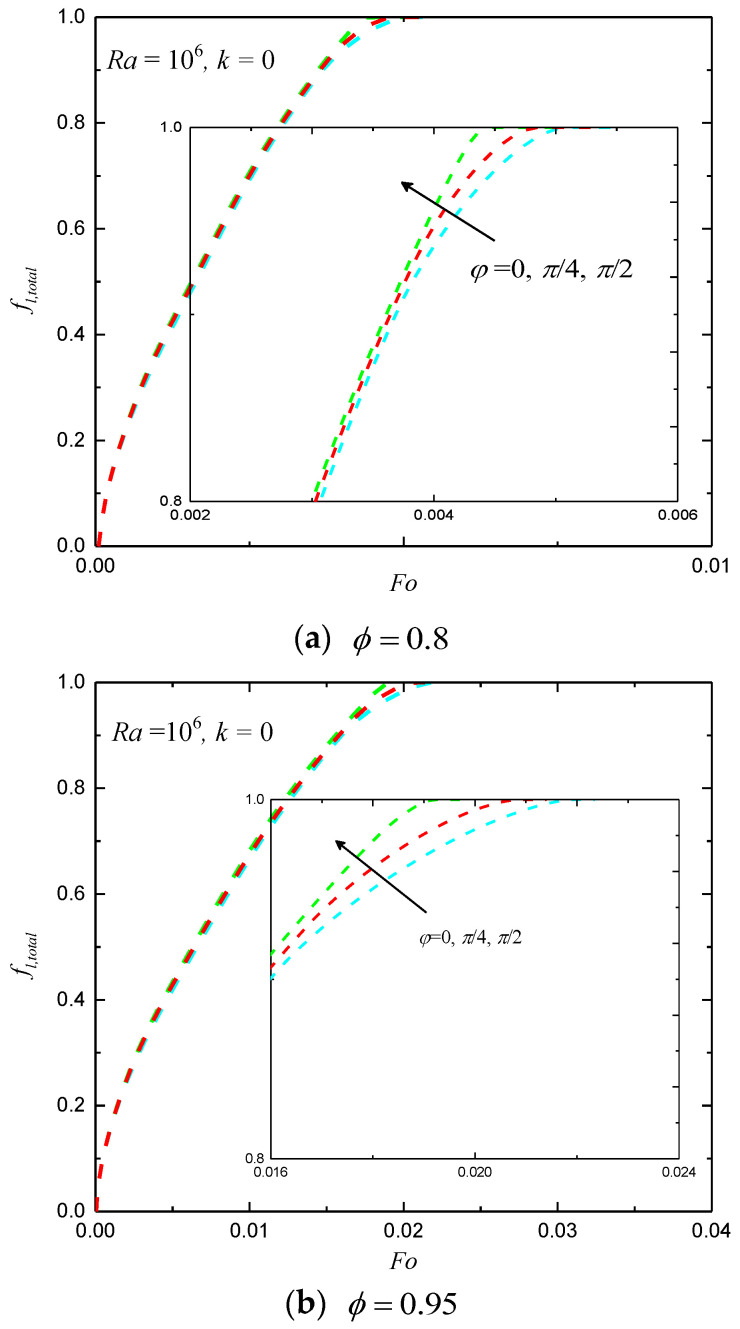
The total liquid fractions for different values of φ under non-uniform thermal boundary conditions.

In Figure 5, the total liquid fractions for ϕ=0.8 and 0.95 under non-uniform ($T=T_{h}+\Delta T\sin\left(2\pi y/L+\pi /4\right)$ at t=0) and uniform ($T=T_{h}$ at t=0) thermal boundary conditions are presented. One can observe that the melting rate of the uniform case is only a little faster than that of the non-uniform case with the given parameters, as the characteristics temperatures are equal for the cases considered. In Figure 6, the total liquid fractions for different φ with $Ra=10^{6}$ and k=0 under non-uniform thermal boundary conditions are shown. It can be observed that the melting rate increases as φ increases from 0 to π/2. As shown in Figure 5 and Figure 6, it seems that the effects of the phase deviation on the total liquid fraction are not very strong. This is because the average temperature of the left wall (with sinusoidally varying temperature) equals a constant.

**Figure 7:**
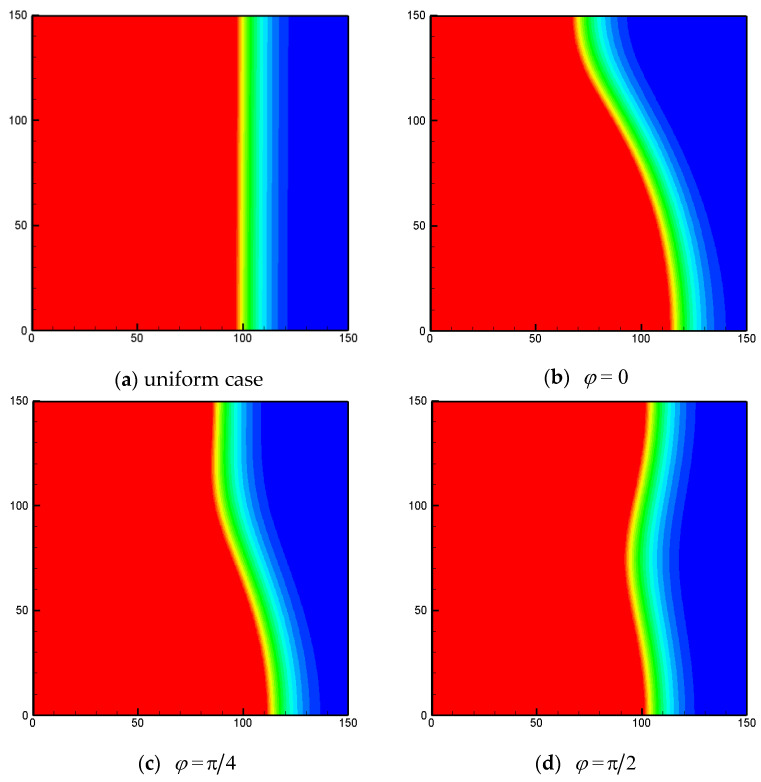
The liquid-fraction fields for different values of φ with *Φ* = 0.8 under non-uniform thermal boundary conditions (*Fo* = 0.0025, *Ra* = 10^6^, *k* = 0, *N_x_* × *N_y_* = 150 × 150).

**Figure 8:**
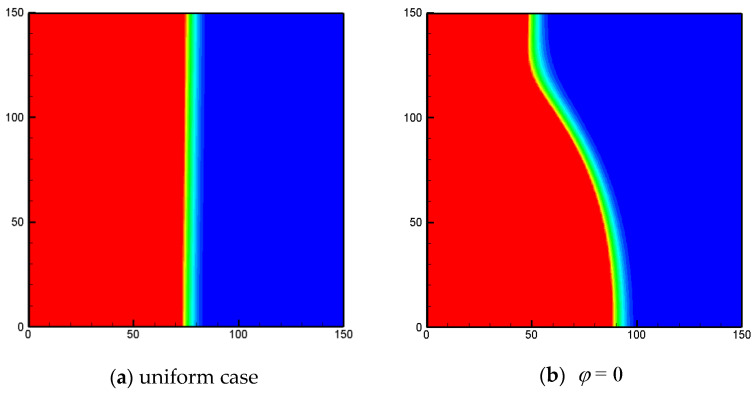

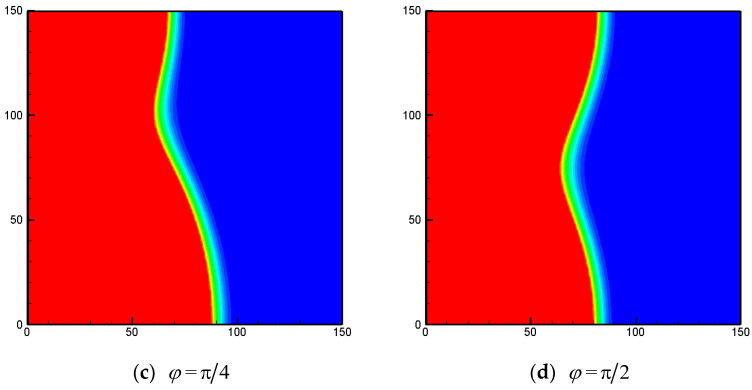
The liquid-fraction fields for different values of φ with *Φ* = 0.95 under non-uniform thermal boundary conditions (*Fo* = 0.0065, *Ra* = 10^6^, *k* = 0, *N_x_* × *N_y_* = 150 × 150).

In Figure 7 and Figure 8, the liquid-fraction fields for different values of φ with ϕ=0.8 and 0.95 under non-uniform thermal boundary conditions are shown. As mentioned above, the effects of the phase deviation on the total liquid fraction are weak. However, in Figure 7 and Figure 8 it can be clearly observed that the melting process can be significantly affected by the phase deviation. For the cases under uniform heating (Figure 7a and Figure 8a), the melting front is almost parallel to the vertical walls, as the conduction effect dominates the heat-transfer process. For the cases under sinusoidal temperature boundary conditions, the phase interface is in a bending shape. This is because under the non-uniform thermal boundary condition, the convection effect in the related region is much stronger than that in the rest of the region. As shown in the figures, for 0<φ<π/2, the convective effect near the bottom wall is stronger and the melting front moves faster near the bottom wall. Obviously, this feature is rather valuable for practical LHS applications, as it offers a possible tool for controlling the melting front.

### 4.2. Effects of the Periodicity Parameter

**Figure 9:**
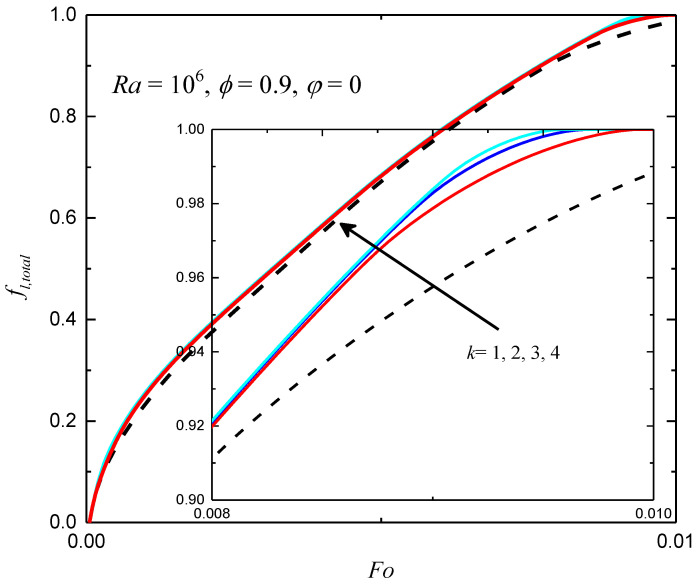
The total liquid fractions for different values of φ under non-uniform thermal boundary conditions (*Ra* = 10^6^, *Φ* = 0.9 and *φ* = 0).

**Figure 10:**
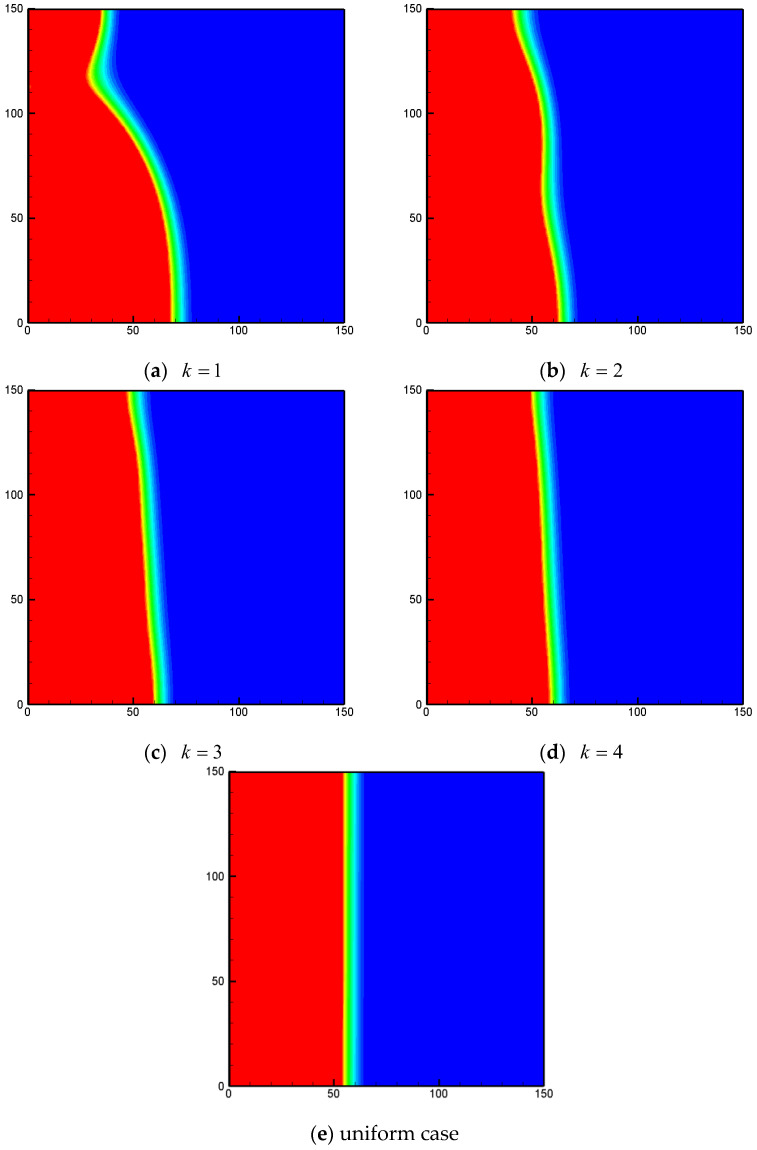
The liquid-fraction fields for different values of k under non-uniform thermal boundary conditions (*Fo* = 0.002, *Ra* = 10^6^, *Φ* = 0.9, *φ* = 0, *N_x_*× *N_y_* = 150 × 150).

In this subsection, the effects of the periodicity parameter k on the performance of heat transfer are studied. In Figure 9, the total liquid fractions for different values of the periodicity parameter k with $Ra=10^{6}$, ϕ=0.9 and φ=0 are shown. As presented in the figure, the effects of the periodicity parameter on the total liquid fraction are weak. As k increases, the melting rate slightly increases, and approaches that of the uniform heating case. The liquid-fraction fields for different k at Fo=0.002 under non-uniform thermal boundary conditions are presented in Figure 10, and one can observe that the melting front can also be affected by the periodicity parameter. As k increases to 4, the melting front is almost parallel to the vertical walls, which is similar to the situation of the uniform case.

## 5. Conclusions

An MRT-LB method in conjunction with the enthalpy approach was constructed for simulating convection melting in metal foam under sinusoidal temperature boundary conditions. The effects of the porosity, the phase deviation, and the periodicity parameter on the heat-transfer characteristics were investigated. The main conclusions are listed as follows:

(1) The melting rate decreases as ϕ increases. The influence of the porosity on the melting rate is induced by two factors: one is that the mass of the metal foam decreases as the porosity increases, which reduces the effective thermal conductivity; the other is that the mass of the PCM increases as the porosity increases, which results in melting time augmentation.
(2) The melting rate increases as the phase deviation increases from 0 to π/2. Although the effects of the phase deviation on the melting rate (total liquid fraction) are weak, the melting front can be significantly affected by the phase deviation.
(3) The effects of the periodicity parameter on the total liquid fraction are weak. However, the melting process can also be affected by the periodicity parameter. The above results provide a valuable reference for practical applications of LHS systems.
